# Arabidopsis MAP-Kinase 3 Phosphorylates UDP-Glucose Dehydrogenase: a Key Enzyme Providing UDP-Sugar for Cell Wall Biosynthesis

**DOI:** 10.1007/s11105-018-1130-y

**Published:** 2018-12-08

**Authors:** Michael Kohlberger, Theresa Thalhamer, Richard Weiss, Raimund Tenhaken

**Affiliations:** 1 Department of Biosciences, Plant Physiology, University of Salzburg, Hellbrunner Str. 34, 5020 Salzburg, Austria; 2 Department of Biosciences, Allergy and Immunology, University of Salzburg, Hellbrunner Str. 34, Salzburg, Austria

**Keywords:** Nucleotide sugar biosynthesis, Enzyme regulation, MAPK, UDP-glucose, UDP-glucuronic acid

## Abstract

The enzyme UDP-glucose dehydrogenase (UGD) competes with sucrose-phosphate synthase for the common photosynthesis product UDP-glucose. Sucrose-phosphate synthase is part of a pathway for the export of sucrose from source leaves to neighboring cells or the phloem. UGD is a central enzyme in a pathway for many nucleotide sugars used in local cell wall biosynthesis. Here, we identify a highly conserved phosphorylation site in UGD which is readily phosphorylated by MAP-kinase 3 in Arabidopsis. Phosphorylation occurs at a surface-exposed extra loop in all plant UGDs that is absent in UGDs from bacteria or animals. Phosphorylated sucrose-phosphate synthase is shifted to an inactive form which we did not measure for phosphorylated UGD. Plant UGDs have an extra loop which is phosphorylated by AtMPK3. Phosphorylation is not causing a reduction of UGD activity as found for the competitor enzymes and thus sets a preference for maintaining UDP-sugars at a constant level to prioritize cell wall biosynthesis.

## Background

The conversion of CO_2_ into carbohydrates by green plants is the basis for our environment and the primary food chain. Fossil energy is only available in limited amounts and thus fixation of CO_2_ in energy rich compounds by plants is of particular importance. This includes the use as feed, as combustible material, and as a raw product for future biofuels. The latter two uses rely heavily on plant cell walls (reviewed (Pauly and Keegstra 2008)).

The partitioning of assimilates between the competing pathways at the cellular and at the organ level is of major importance for the energy conversion of sunlight into useful chemicals. This partitioning is influenced by the regulation of a complex developmental program that decides, whether to use carbohydrates for storage (for example, in seeds) or whether to invest them in plant growth, tightly connected with the biosynthesis of new cell wall polymers.

Many enzymes in carbohydrate metabolism are regulated at several levels, including transcriptional changes, feedback inhibition by metabolites, redox modifications, and posttranslational modifications such as phosphorylation and glycosylation (Sulpice et al. 2014; Dietz and Hell 2015; Huber and Huber 1996).

**Fig. 1:**
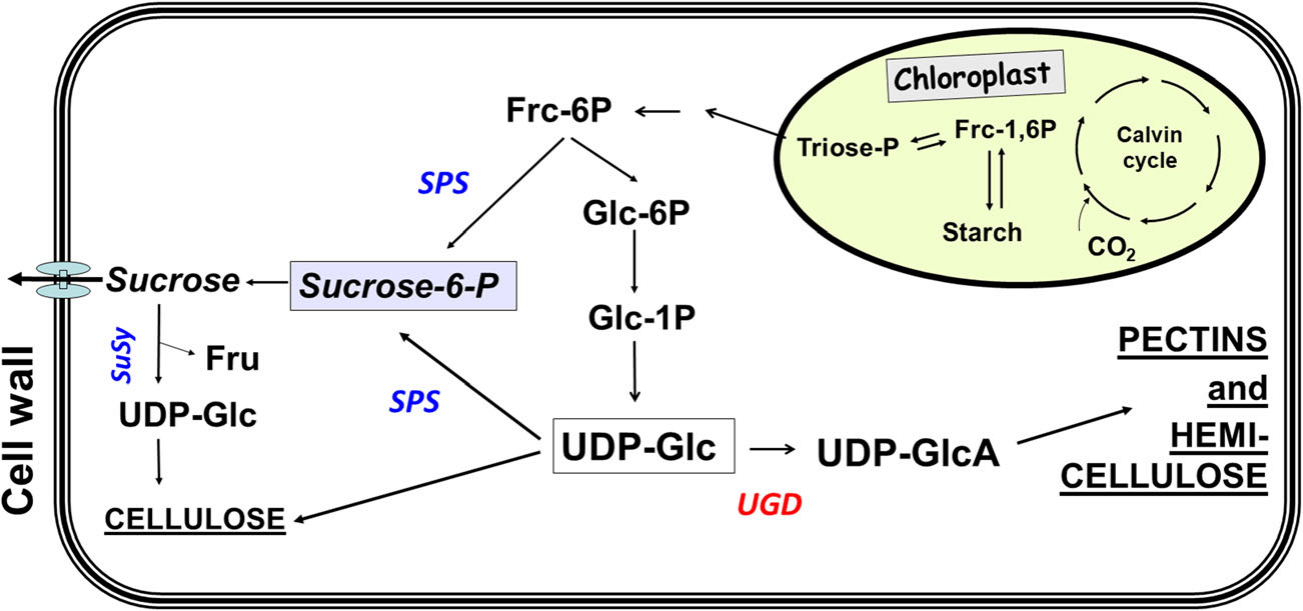
Cellular pathway showing the central role of UDP-Glc in carbon partition between exported sucrose and formation of UDP-GlcA as important precursors for pectins and hemicelluloses

As outlined in the pathway in Fig. 1, UDP-glucose is a central intermediate in the partitioning of carbohydrates (Reboul et al. 2011; Seifert 2004; Bar-Peled and O'Neill 2011). On the one hand, it is the direct precursor of sucrose in the enzymatic reaction catalyzed by sucrose-phosphate synthase (SPS) and on the other hand, it is the direct precursor for the synthesis of cellulose and further cell wall polymers via the enzyme UDP-glucose dehydrogenase (UGD). This enzyme is encoded by a four-member gene family in Arabidopsis (Klinghammer and Tenhaken 2007). UGDs catalyze the irreversible 4-electron oxidation of UDP-glucose (UDP-Glc) into UDP-glucuronic acid (UDP-GlcA), concomitant with the production of two molecules NADH from NAD^+^. UDP-GlcA is subsequently converted into the nucleotide sugars of galacturonic acid for the synthesis of pectins, and of xylose and arabinose for the synthesis of hemicelluloses (Reiter 2008). In the model plant *Arabidopsis thaliana*, roughly 50% of the cell wall biomass of leaves is derived from the common precursor UDP-GlcA (Zablackis et al. 1995). As this nucleotide sugar is mainly used for cell wall biosynthesis (and to a small extent for protein glycosylation and conjugates of secondary metabolites), the formation from UDP-Glc is tightly regulated.

The enzyme sucrose-phosphate synthase (SPS) is the major competitor of UGD for the substrate UDP-Glc (Fig. 1). It is long known to be regulated by several posttranslational modifications, resulting in a switch between active and partially inactive forms of the enzyme (Huber and Huber 1996; Stitt et al. 1988). The phosphorylation of Ser_158_ of the spinach SPS occurs mainly in the dark and inactivates the enzyme (Toroser et al. 1999), whereas a second phosphorylation at Ser_424_ leads to an activation (Toroser and Huber 1997). Beside these direct modifications of SPS, allosteric activation or inhibition by metabolites (e.g., glucose-6-P as an activator; P_i_ as an inhibitor) has been identified as an additional level of regulation. SPS isolated from leaves, which were incubated for 30 min in the dark, mainly exists in the low affinity form. The studies on SPS mostly consider the regulation of carbohydrates but neglect the interaction with the biosynthesis of UDP-sugars for cell wall polymers. A strong activity of SPS in the dark will likely lower the concentration of UDP-Glc, when photosynthesis is inactive and carbon availability is dependent on the degradation of transient starch. When plants were stressed by an extended dark period, the degradation rate of transitory starch is adjusted to supply energy until the end of the dark period (Smith and Stitt 2007).

Here, we report on the phosphorylation of a serine residue in a plant-specific loop in UGD by MAP-kinase 3 and investigate the biochemical role of this modification.

## Materials and Methods

### Expression of Recombinant Proteins

The expression construct for Arabidopsis UGD3 was cloned by fusing the open reading frame of UGD3 (At5g15490) to a His-tagged maltose binding protein. The His-MBP-UGD3 is expressed at far higher levels compared to His-AtUGD3 alone (Klinghammer and Tenhaken 2007) and has the same *K*_m_ values for NAD^+^ and UDP-Glc as described (Klinghammer and Tenhaken 2007). Therefore, His-MBP-UGD3 is used in all experiments present here. Bacteria were grown with rigorous shaking to an OD_600nm_ of 0.6. Thereafter, the culture was cooled down to 18 °C and 0.5 mM IPTG was added to the culture and further grown for 16 h. Recombinant UGD was purified on a Protino-TED column (Machery-Nagel, Düren Germany) using the protocol of the manufacturer. The buffers (LEW and EB) were supplemented with 10% glycerol and 0.2 mM NAD^+^ to maintain full enzyme activity of UGD during purification. Bacteria were collected by centrifugation and resuspended in LEW-buffer. Lysozyme (1 mg mL^−1^) and PMSF (1 mM) were added and cells were frozen at − 20 °C to gently break the cells. The cells were thawed on ice and incubated for 30 min. The cell suspension was treated with ultrasonic power (1 kJ in total) with 15-s pulses and 15-s cooling breaks. After purification, glycerol was added to a final concentration of 20% and active UGD was snap frozen in small aliquots and stored at − 70 °C until use.

To make use of the tobacco transient leaf expression system, the open reading frame of AtUGD3 was cloned into pGreen0229 with a C-terminal StrepTag (8 amino acids). The UGD-gene was expressed under the control of the CaMV35S-promotor and translated to a 54 kDa protein. The StrepTag was used to purify the tagged protein from tobacco leaf crude extracts, using the Macroprep Step-Tactin matrix (IBA, Göttingen, Germany).

Arabidopsis MAP-kinase 3 (AtMPK3) was expressed as a GST-fusion protein from plasmid pGEX4Z-AtMPK3-GST kindly provided by Lennart Eschen-Lippold (IBP Halle, Germany). *E.coli* cultures were grown to OD_600nm_ of 0.6 and cooled down to 18 °C. Induction of recombinant AtMPK3 was started by adding of 0.5 mM IPTG overnight. Recombinant protein was purified on GST-agarose (Biontex) according to the protocol provided by the manufacturer.

To obtain an active AtMPK3, it is necessary to phosphorylate the MAP-kinase by a suitable MAP-kinase-kinase (MKK). Constitutively active parsley MKK5-DD (Lee et al. 2004; Strehmel et al. 2017) was expressed as a His-tagged fusion protein from plasmid pJC40-PcMKK5-His kindly provided by Lennart Eschen-Lippold (IBP Halle, Germany). The group in Halle has successfully used the parsley MKK5 to phosphorylate AtMPK3. *E.coli* cultures were grown to OD_600nm_ of 0.6 and cooled down to 18 °C. Induction of recombinant PcMKK5 was started by adding 0.5 mM IPTG overnight and purified on a Protino-TED column (Machery-Nagel), using the standard protocol of the manufacturer.

### Enzyme Assays

Phosphorylation of AtUGD3 was achieved by the activity of two kinases. A parsley MKK5-clone, constitutively active by an exchange of two serine/threonine residues against two aspartates, is phosphorylating recombinant AtMPK3 at its active center. The active AtMPK3 then phosphorylates the UGD-enzyme in the presence of ATP. The phosphorylation buffer consists of 50 mM Tris/Cl pH 7.5, 5 mM MgCl_2_, 2 mM MnCl_2_, 1 mM ATP, 1 mM DTT, and 0.2 mM NAD^+^. AtMPK3 and PcMKK5 were added at 1/10 of the amount of AtUGD3. The assay was incubated at 30 °C for 5–30 min.

Controls with heat-inactivated PcMKK5 and AtMPK3 were performed and added to UGD enzyme assays.

Enzyme assays for UGD were performed in 50 mM Tris/Cl pH 8.7, 1 mM NAD^+^, and 0.5 mM UDP-Glc. The substrate concentrations were varied in some experiments for enzyme kinetics as indicated in the text. The increase in NADH was measured at 340 nm and used to calculate the enzyme activity.

### Generation of Monoclonal Antibodies Against UGD-Peptides

The peptide 17 (CHPLHLQPMSPTTVKQV) was synthesized at GeneCust, Evry, France with (17P) and without (17) the Ser-phosphorylation. Peptides were dissolved in dry DMSO to a final concentration of 10 mg mL^−1^ and coupled to cationized bovine serum albumin (cBSA) or ovalbumin (OVA) as carriers. Briefly, 50 μL of peptide were mixed with 250 μL of cBSA or ovalbumin (2.5 mg mL^−1^) and 33 mL of 1 M MES buffer, pH 4.7. Subsequently, 0.7 mg of EDC cross-linker was added and the reaction was incubated over night at 4 °C. After dialysis against PBS, peptide conjugates were stored at − 20 °C until further usage. Mice were immunized 3 times in 2-week intervals with 10 μg of BSA-17 or BSA-17P adsorbed to alum (Alu-Gel-S, Serva). Mice tested as seropositive by ELISA using OVA-17 and OVA-17P as antigen were sacrificed and immune spleen cells were fused with P3–X63–Ag8 myeloma cell line by standard methods. Cells were incubated in Optimem I (supplemented with 5% FBS, 1 × Pen/Strep, 50-100 U/mL hIL-6, 1 × Azaserin-Hypoxanthin) at 37 °C, 95% relative humidity, 5% CO_2_ for 10 days. At the 5th day of incubation 1-mL fresh medium was added. At day 10, supernatants were analyzed for anti-17 and anti-17P IgG antibodies by ELISA using the respective peptides coupled to OVA. Hybridomas from positive wells were cloned by limiting dilution.

### SDS-PAGE and Western Blot

Proteins were separated on 10% acrylamide gels in a Biorad mini gel apparatus. For Western blots, the proteins from the gel were transferred onto PVDF-membrane (Millipore) by tank blotting electro transfer. Western blots were performed by first blocking free binding sites on the membrane with 5% milk powder in TBST for 1 h. The blots were washed with TBST and incubated with the monoclonal antibodies, diluted 1:50 in TBST-5% BSA overnight in a cold room. Blots were washed 3 times for 10 min each with TBST and thereafter incubated with a secondary anti-mouse-HRP antibody (Santa Cruz SC-2005; 1:7500 dilutions in TBST with 0.5% BSA) for 1 h at room temperature. After washing the blot three times 10 min each in TBST, the chemiluminescence reaction was started. Images were recorded on a Fuji-LAS3000 cooled camera system.

### PhosTag Gels

A 5% acrylamide PhosTag gel was prepared with 50 μM PhosTag chelator and 200 μM MnCl_2_ according to the protocol of the manufacturer (Wako-Chemicals, Japan). The concentrations of acrylamide, PhosTag chelator and MnCl_2_, were chosen from the results of an optimization process. The low acrylamide concentration is necessary as the MBP-UGD fusion protein has a Mr. of about 97 kDa. The gels (minigel size 8 cm height) were run at 100 V for100 min at 4 °C.

## Results

### Plant UGDs Have a Conserved Insertion Sequence

**Fig. 2:**
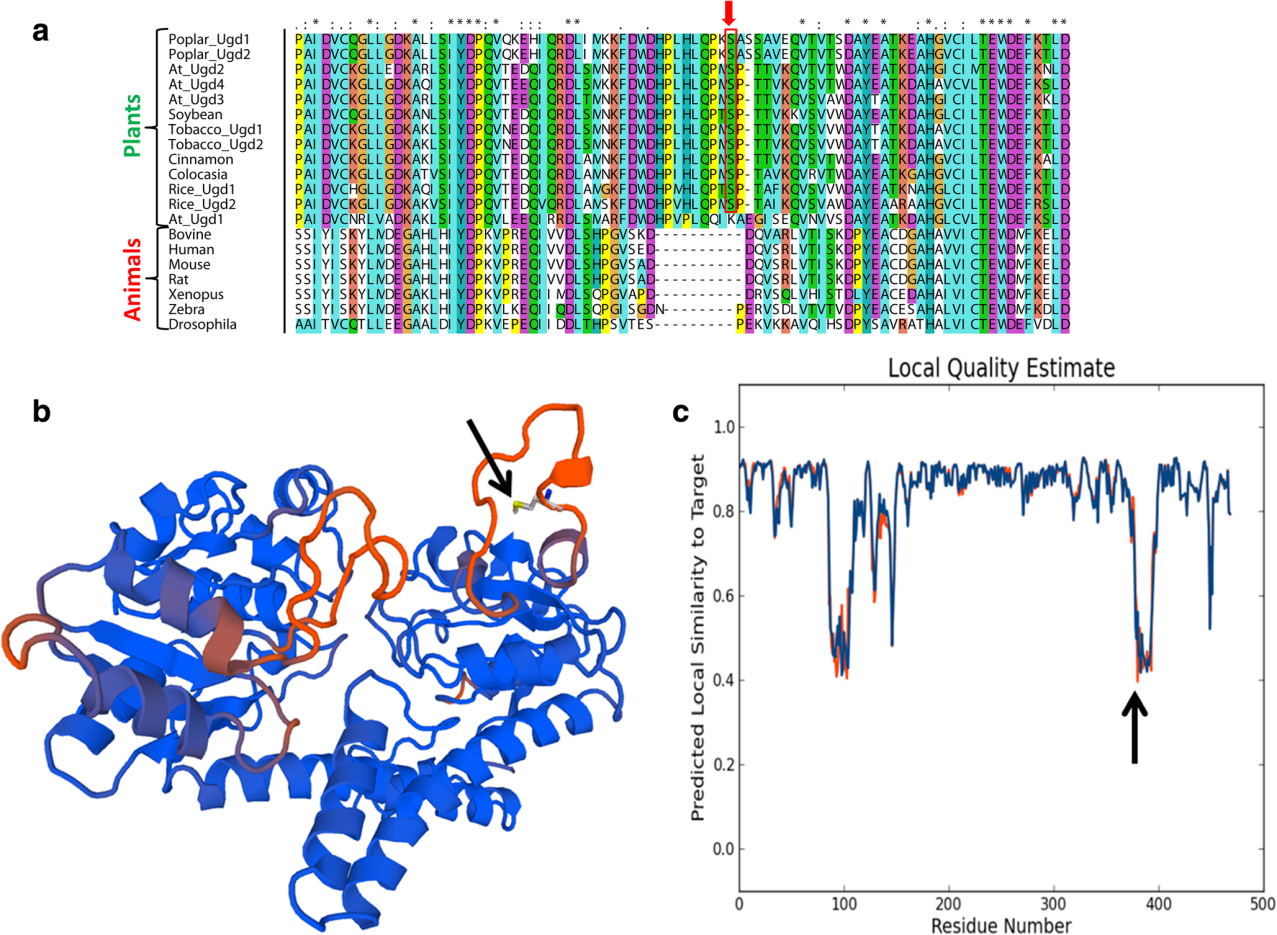
Alignment of selected sequence for animal and plant UGDs. **a** A segment is shown to highlight a 10 amino acid insertion in plant UGDs. The serine_393_ residue being phosphorylated by AtMPK3 is boxed. Fully conserved residues are marked with an asterisk “*” in the consensus line above. Colon “:” means fully conserved residues of strong groups and full stop “.” means fully conserved residues of weaker groups as defined in the CLUSTAL software. **b** The sequence of AtUGD3 was modeled using the human UGD (4EDF) as template by the SWISS MODELL software package. Red color indicates regions with lower local similarity. The Serine_393_ is highlighted by an arrow and lies in a surface exposed linker region between conserved secondary structures. The degree of predicted local similarity between the human and the Arabidopsis sequence is shown in (**c**)

The enzyme UGD is present in almost all pro- and eukaryotes with a few exceptions in secondarily reduced genomes like baker’s yeast. By comparing plant and animal UGD amino acid sequences, it becomes obvious that all plant sequences have a small insertion of about 10 amino acids around position 390 (Fig. 2a). The insertion displays a motif of –P-X-S-P- which is the prototypic phosphorylation site of MAP-kinases (Hoehenwarter et al. 2013). In Arabidopsis, UGD2, UGD3, and UGD4 share an identical insertion sequence, whereas UGD1 still has an insertion of the same length but differs in sequence. In particular, the serine residue is lacking (Fig. 2a). The serine S_393_ within the –P-X-S-P- motif was found to be phosphorylated in phosphoproteomic studies (Benschop et al. 2007; van Bentem et al. 2008) confirming that this Ser-residue is phosphorylated in vivo at least in certain conditions.

UGD is a quite conserved enzyme in all organisms. This allowed us to take the crystal structure of the human UGD (4EDF) to model the Arabidopsis sequence and to identify the position of the insertion in the protein structure. The insertion extends a surface exposed linker between a conserved β-barrel and a close by α-helix (Fig. 2b). The high degree of predicted structural similarity is shown in Fig. 2c. There are two regions with a lower local similarity score and the Ser393 is exactly in the middle of one of these low similarity regions. This underlines the observation that plant UGD sequences differ in this segment from animal UGDs.

### Monoclonal Antibodies Against Phosphorylated UGD

We used the insertion sequence to synthesize the immunogenic peptides. One version of the peptide contained the Ser393 in a phosphorylated version. The peptides were coupled to cationize BSA and used to immunize mice. Hybridoma clones, showing interaction with either one of the peptides, were selected and enriched to obtain single mAB expressing cell lines. We obtained mAB from cell lines interacting with either both forms of the UGD protein or a few lines, which express a mAB that only interacts with the phosphorylated form of the peptide. A single line, interacting most strongly with UGD or UGD-P, was used in further experiments. In crude plant extracts, the antibodies failed to detect specific bands in western blots. The amount of the UGD protein is obviously too low to allow specific detection of the UGD protein in crude extracts. The detection is further complicated by the molecular weight of the UGD protein, which is very similar to that of the large subunit of the abundant Rubisco enzyme. In summary, the mABs are a useful tool to discriminate between UGD and UGD-P but they cannot be used for experiments in plant crude extracts to quantify the phosphorylation level of UGD.

**Fig. 3:**
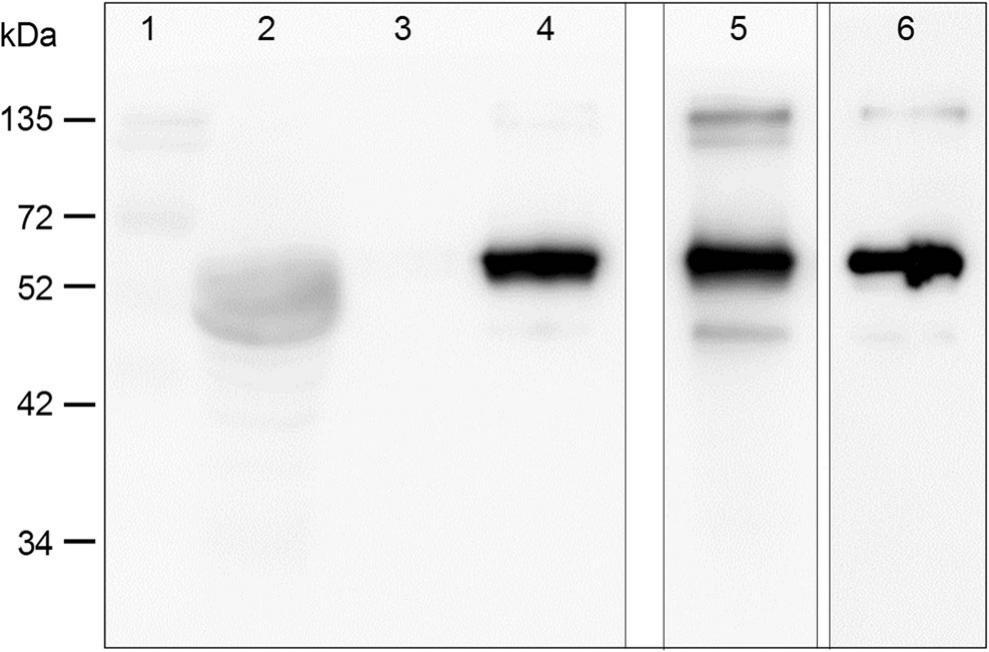
Western blot with tobacco extracts obtained from transient overexpression of UGD. Lanes 1–4 were incubated with a UGD-P specific mAB. Lane 1, marker; 2, crude extract depleted from Strep-UGD; 3, washing step StrepTag purification; 4, eluate. Lane 5, eluate but incubated with UGD-mAB. Lane 6, eluate but incubated with StrepTag-POD

Next, we tested whether UGD is phosphorylated in plants. Therefore, we constructed a binary plasmid that allows transient expression of Arabidopsis UGD in tobacco leaves. A C-terminal StrepTag was added to the open reading frame of UGD to allow purification of the recombinant UGD protein from leaf crude extracts. As shown in Fig. 3, UGD is expressed in tobacco leaves can be purified via the StrepTag. Both mAB recognize the UGD (54 kDa) in tobacco suggesting that a major portion of the recombinant UGD is phosphorylated in vivo.

### AtMPK3 Phosphorylates UGD

**Fig. 4:**
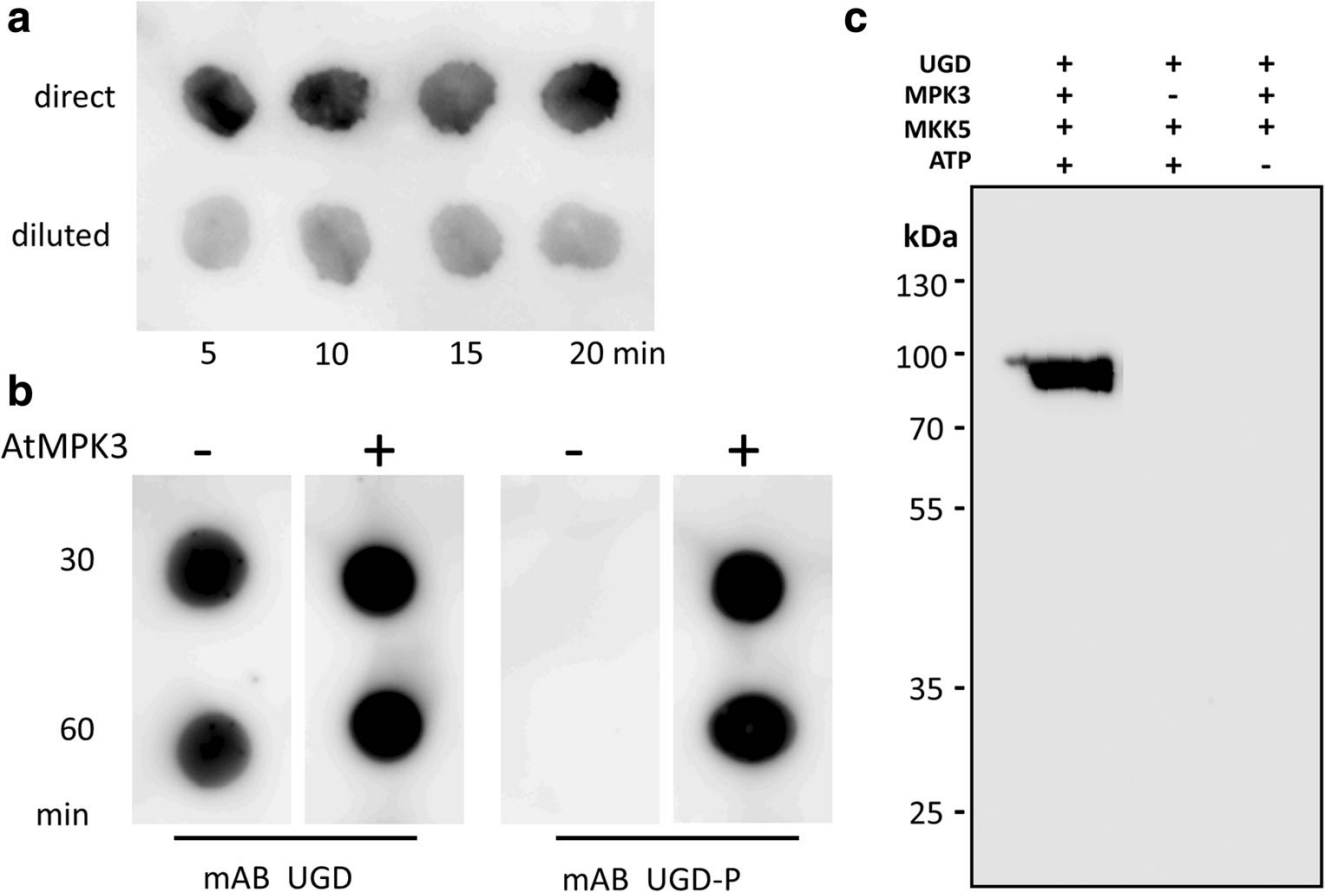
**a** Time kinetic of UGD phosphorylation by AtMPK3. A phosphorylation assay was set up and aliquots were removed after 5, 10, 15, or 20 min. Upper row, undiluted sample; lower row 1:3 diluted sample. **b** Specificity of mAB UGD-P. Samples from a phosphorylation assay were spotted on a nitrocellulose membrane. In control assays (AtMPK3), the two kinases were heat inactivated before they were added to the phosphorylation assay. The UGD mAB recognizes both forms of UGD whereas the mAB UGD-P only interacts with phosphorylated UGD. **c** Western blot with mAB-UGD-P showing the specificity of the phosphorylation reaction. Only the full assay in the left lane leads to a strong western blot signal as predicted. Controls, in which either the MPK3 protein in missing (middle lane) or the kinase substrate ATP was left out (right lane), did not show any signal with the mAB-UGD-P specific antibody

The phosphorylation motif –P-X-S-P- is a prototype motif of MAPK. We therefore focused on MAPKs as likely candidates being involved in phosphorylation of UGD. One possible experiment to narrow down the number of candidate kinases is the use of the yeast-two-hybrid system. Specific MAPK isoforms may bind to their substrates strong enough that this interaction can be identified by yeast-two-hybrid screenings (Yang et al. 1992). We therefore performed a yeast-two-hybrid study with 20 Arabidopsis MAPK-clones as prey and the UGD as bait to test for possible interaction between a MAPK and a putative substrate. Unfortunately, none of the 20 MAPK showed any interaction with the UGD protein. The most prominent MAPK in Arabidopsis are AtMPK3, AtMPK4, and AtMPK6, which have been studied well (Morris 2001; Rodriguez et al. 2010). Out of these 3 MAPK, only AtMPK3 was able to phosphorylate UGD in vitro. We therefore focused on AtMPK3 as the phosphorylating kinase of UGD. To obtain active AtMPK3 a mixture of AtMPK3 as well as an activating MAP-kinase kinase (MKK) is needed. We expressed AtMPK3 and an upstream activating kinase MKK5 from parsley as recombinant proteins. By combining the two kinases with UGD as substrate, we observed a rapid phosphorylation of the UGD protein in vitro. Controls, in which either MPK3 or ATP is missing, did not phosphorylate UGD3 (Fig. 4c). Furthermore, the mAB-UGD-P only recognizes the phosphorylated form of MBP-UGD with a molecular weight of approx. 97 kDa. This allowed us to perform phosphorylation studies on simple dot blots. There, already 5 min after mixing all components together, most of the UGD protein is already phosphorylated (Fig. 4a). The signal in the western blot remains constant after 5 min for the next 30 min, indicating that UGD-phosphorylation is almost complete after few minutes in our assay. In our assay conditions, UGD is a well-accepted substrate of AtMPK3. The specificity of the mAB-UGD and mAB-UGD-P could be confirmed by this in vitro phosphorylation assay. As shown in Fig. 4b, the mAB-UGD recognizes the UGD protein independent of the phosphorylation status. When using the mAB-UGD-P, it became evident that only the phosphorylated form is recognized by this antibody. Controls with heat-inactivated AtMPK3 in the phosphorylation assay gave no signal with the mAB-UGD-P. The strength of the signal is in agreement with Fig. 4a that the phosphorylation occurs very rapidly.

### Properties of Phosphorylated UGD

**Fig. 5:**
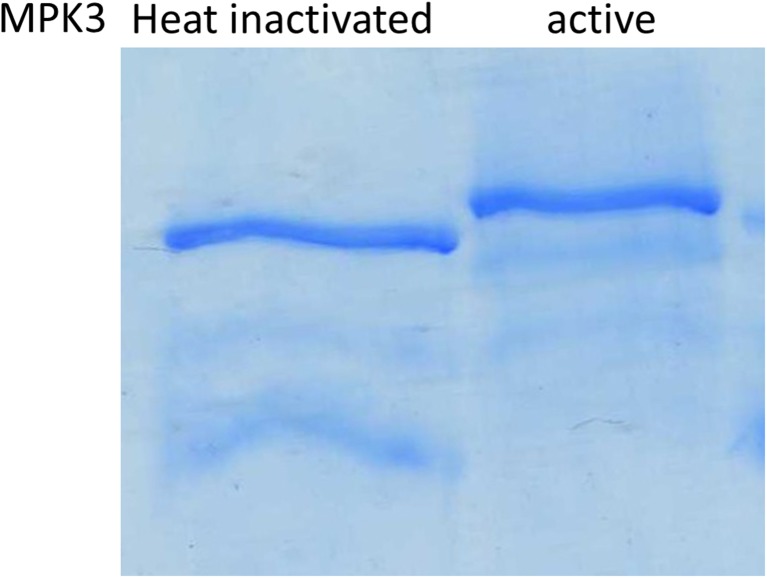
PhosTag SDS-Page with unphosphorylated UGD (heat inactivated kinases) and phosphorylated UGD. A 5% gel was used. Due to the interaction with the immobilized chelator-Mn^2+^ complexes UGD-P is more retarded than UGD in controls

The experiments so far do not rule out that UGD is phosphorylated multiple times. To test this, we established PhosTag acrylamide gels which allow a physical separation of phosphorylated proteins by retardation with immobilized chelated metal ions, using Mn^2+^ in our case. Multiple phosphorylations of proteins typically result in a laddering of phosphoproteins as each phosphate group contributes to retardation via Mn^2+^-phosphate interaction. The UGD-band is easily identified by the Coomassie stain as the assay contains about 80% MBP-UGD protein, ~ 10% AtMAPK3 and ~ 10% MKK5. Furthermore, the molecular weight of the MBP-UGD is far higher than that of the kinases. The Coomassie stained gel in Fig. 5 shows a single and only slightly retarded band in the gel, suggesting that the UGD protein is only phosphorylated once. This is consistent with the finding that phosphoproteomic studies identified Ser_393_ several times (Benschop et al. 2007; van Bentem et al. 2006). Controls in which AtMPK3 and PcMKK5 were heat inactivated before addition of the UGD as substrate do not show any retardation in PhosTag gels (Fig. 5).

**Fig. 6:**
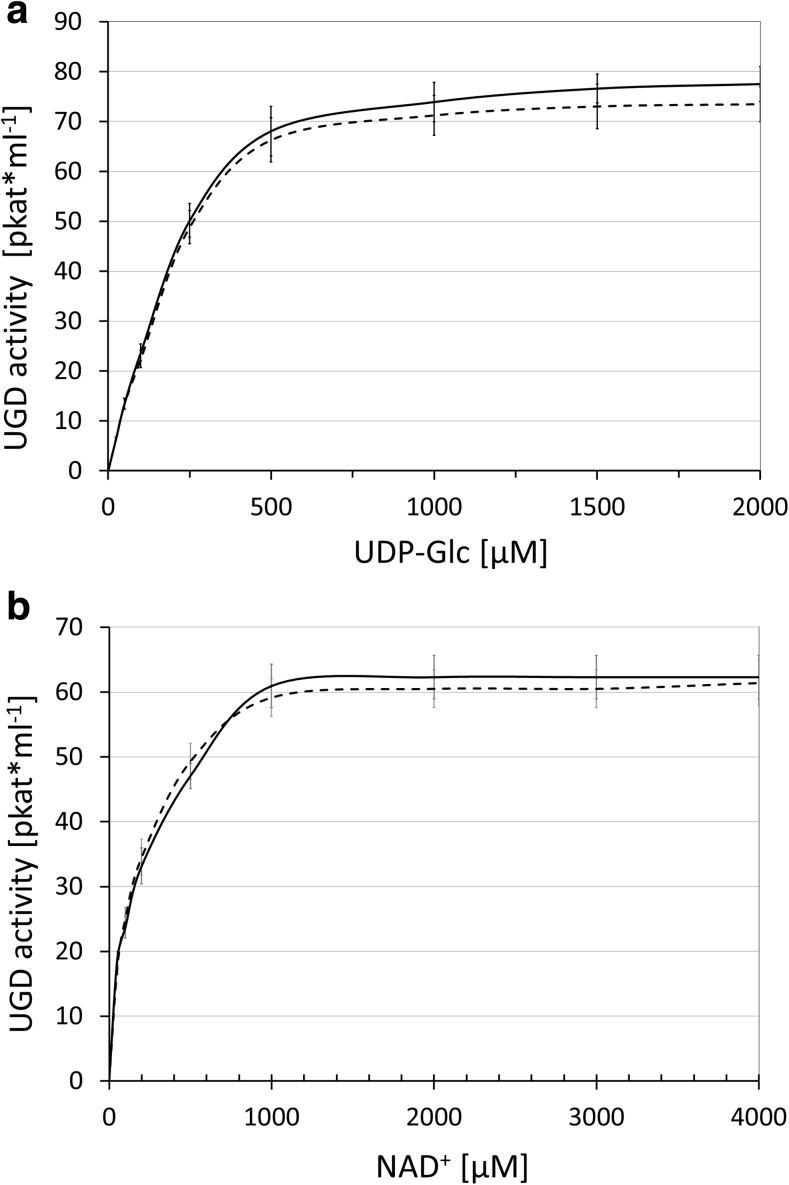
Kinetic of UGD and UGD-P. **a** The *K*_m_ for UDP-Glc was determined for both forms of UGD showing the same *K*_m_ and well as the same activity (*V*_max_). **b** The *K*_m_ for NAD^+^ was determined for both forms of UGD showing the same *K*_m_ as well as the same activity (*V*_max_). The dotted line represents the unphosphorylated control; the solid line the phosphorylated UGD-P

As the experiments clearly show that UGD is largely phosphorylated within a few minutes, we decided to use the phosphorylated UGD directly for enzymatic assays. Control assays contained the same amount of AtMPK3 and PcMKK5 but heat inactivated. Though our hypothesis was that phosphorylated UGD differs in activity from the non-phosphorylated form, we could not find any difference in the substrate affinities for UDP-Glc or NAD^+^ (Fig. 6). The velocity of UDP-Glc conversion was also the same for both forms of UGD.

## Discussion

The enzyme UGD occurs as a four-member gene family in Arabidopsis, which is also typical for other plants. Three out of the four members contain a classical MAP-kinase motif comprised of –P-M-S-P-. Though we did all of our experiments with AtUGD3, we strongly believe that isoforms 2 and 4 are also phosphorylated at the same position. Trypsin digestion of UGD2, UGD3, and UGD4 release the identical phosphorylated peptide which makes it difficult to predict, which isoform of UGD was the origin. We tested directly in vitro phosphorylation of UGD using an activated AtMPK3 from Arabidopsis. The UGD protein is readily phosphorylated at S_393_. The trypsin-derived phosphopeptide covering S_393_ has been detected in phosphoproteomic studies before (Hoehenwarter et al. 2013; Benschop et al. 2007; van Bentem et al. 2008). This argues against a random phosphorylation event but strongly supports a specific and consistent phosphorylation at S_393_ under different environmental conditions.

Phosphorylation of enzymes is often involved in modifying the activity to allow flexible regulation at a posttranslational level (Lee et al. 2015; Halford and Hey 2009). In plant growth, UGD and sucrose-phosphate synthase compete for the same substrate UDP-Glc. Both enzymes serve different cellular functions and flexible regulation of their activities allows the plant cell to promote or inhibit either one. The synthesis of sucrose allows the export of carbohydrate to neighboring cells and the phloem long-distance transport, whereas usage of UDP-Glc by UGD will shift activated nucleotide sugars irreversibly into a pool of cell wall precursors (Klinghammer and Tenhaken 2007). This is in particular useful when the plant cell is still growing and needs matrix polysaccharides to fill up the cell wall material after thinning caused by extension growth. Though the major route for sucrose is often the export out of the cell, sucrose can also be used to synthesize cellulose by cleavage through the enzyme sucrose synthase (Amor et al. 1995). Thus, sucrose formation is not as irreversible in a cellular growth context as is the oxidation of UDP-Glc into UDP-GlcA. We expected a change in the enzymatic activity of phosphorylated UGD but in all experiments performed, we clearly ended up with the same activity of UGD and UGD-P. So, this allows us to draw the conclusion that UGD is readily phosphorylated by AtMPK3 but that this change is not modifying the enzyme activity. If UGD activity is regulated in cellular metabolism, it must occur by other, not yet identified, modes of action. One other possibility could be a relocalization of UGD from the cytoplasm to the Golgi apparatus. The advantage for the cell could be a substrate channeling issue which would allow efficient uptake of UDP-GlcA into the Golgi. We tested whether a functional UGD::GFP fusion protein changes its subcellular localization in a phosphorylation-dependent manner. These experiments gave inconclusive answers but a strong shift of UGD::GFP to the Golgi or other compartments was never observed.

As shown in Fig. 2, all plant UGD sequences show a 10 amino acid insertion around position 390, containing the conserved phosphorylation site, compared to UGDs from animals and bacteria. The insertion is highly conserved in higher plants but can also be found in mosses and at least some green algae. This suggests that the insertion was likely introduced once during evolution and maintained throughout the spreading of land plant species. Arabidopsis UGD1 is an exception as this isoform still contains an insertion of the same length but lacks a serine or threonine for phosphorylation. This particular isoform is expressed in plants typically at lower levels than the other isoforms as visualized for the AtGenExpress Affimetrix chip data with the Genevestigator tool. On the other hand, UGD1 has a far lower *K*_m_ value for UDP-Glc (15 μM (Oka and Jigami 2006)) than the other 3 isoforms (> 100 μM (Klinghammer and Tenhaken 2007)). A high affinity UGD expressed at a lower level might provide basal levels of UDP-sugar for cell wall biosynthesis as it would be a strong competitor of SPS. We therefore asked the question if this pattern of at least one non-phosphorylable UGD is occurring in all plants. GenBank database search clearly reveals that a number of plant species follow this pattern but all of them belong to the family of *Brassicaceae*. This would rather suggest that during a previous genome duplication event of one the UGD-copies mutated at the sequence around position 390 to a UGD1 like sequence which was subsequently maintained within the *Brassicaceae* family.

## Conclusions

The enzyme UDP-glucose dehydrogenase is a competitor with sucrose-phosphate synthase in the cell for the shared substrate UDP-Glc. Whereas sucrose-phosphate synthase is inhibited by phosphorylation during the night, no data were available for UGDs. We identified a surface exposed conserved extra-loop in plant UGDs which contain a MAPK phosphorylation motif. Recombinant AtMPK3 does readily phosphorylate a serine residue of UGD. The phosphorylated UGD retains full enzymatic activity suggesting that the cell sets a preference for UDP-sugars, involved in cell wall biosynthesis.

## Abbreviations

AtMPK3: mitogen-activated protein kinase 3 from Arabidopsis
MAPK: mitogen-activated protein kinase
UGD: UDP-glucose dehydrogenase EC 1.1.1.22
UGD-P: phosphorylated form of UDP-glucose dehydrogenase
mAB UGD: monoclonal antibody recognizing both forms of UGD
mAB UGD-P: monoclonal antibody recognizing specifically the S393-phosphorylated epitope
UDP-Glc: UDP-glucose
UDP-GlcA: UDP-glucuronic acid
